# MGMSN: Multi-Granularity Matching Model Based on Siamese Neural Network

**DOI:** 10.3389/fbioe.2022.839586

**Published:** 2022-03-28

**Authors:** Xin Wang, Huimin Yang

**Affiliations:** ^1^ Huafeng Meteorological Media Group, Beijing, China; ^2^ College of Computer and Software, Nanjing University of Information Science Technology, Nanjing, China

**Keywords:** conversation system, retrieval model, semantic matching, Siamese neural network, multi-granularity

## Abstract

Aiming to overcome the shortcomings of the existing text matching algorithms, in this research, we have studied the related technologies of sentence matching and dialogue retrieval and proposed a multi-granularity matching model based on Siamese neural networks. This method considers both deep semantic similarity and shallow semantic similarity of input sentences to completely mine similar information between sentences. Moreover, to alleviate the problem of out of vocabulary in sentences, we have combined both word and character granularity in deep semantic similarity to further learn information. Finally, comparative experiments were carried out on the Chinese data set LCQMC. The experimental results confirm the effectiveness and generalization ability of this method, and several ablation experiments also show the importance of each part of the model.

## Graphical Abstract

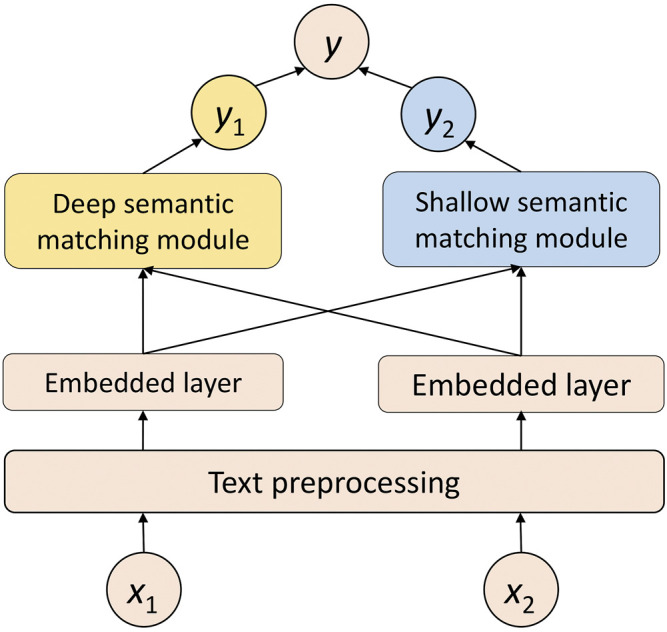

## 1 Introduction

Natural language processing is a hot research topic and a technological frontier in the fields of artificial intelligence and information processing. Understanding and judging language and making corresponding responses are the primary tasks of realizing machine intelligence (Shang et al. (2015); Ma et al. (2021b)). Due to the popularity of smartphones and the development of wireless technology, we are now in the age of social media, and the conversation model has gradually developed into a social mode (Ma et al. (2021a)).

Early interaction systems, such as ELIZA (Weizenbaum (1983)), PARRY (Colby (1981)), and UC (Wilensky (1987)), were conversation models designed to imitate human behavior and pass the Turing test. Despite the impressive success, these conversation models are mainly based on manually customized rules, so they only have limited performance (Lu et al. (2021)). Nowadays, retrieval-based methods are one of the mainstream techniques for constructing conversation models. Generally, a retrieval-based model selects the appropriate response from the predefined corpus based on the input question, for instance, given a question, the retrieval model will calculate the similarity between the input question and each context in the corpus. These matching scores will be sorted, and the response matched by the context with the highest score will be taken as the answer to the input question. The final response quality of a retrieval model is not only affected by the size of the corpus but also depends on the accuracy of sentence similarity calculation. Here, for the latter, it is necessary to analyze and extract the features of the sentence itself and between sentences.

Traditional sentence matching methods are mainly based on statistical characteristics of sentences (Zhang et al. (2021)) or on word embedding (Shen et al. (2018)) to directly calculate the similarity between sentences. But they often ignore the semantic features of sentences, which are not effective in complex situations. With the development of deep learning and its successful application in various fields, using it to mine the deep representation of sentences has attracted more and more attention in sentence matching. Generally, a neural network is used to encode the two statements into sentence vectors, and then the relationship between sentences is determined according to the similarity of the two vectors (Bowman et al. (2015); Yang et al. (2015); Lu et al. (2020b)). However, this kind of framework ignores the lower level interaction between two sentences. The matching–aggregation framework is therefore proposed to match two sentences at the word level and then aggregate the matching information based on the attention mechanism for the final decision. Rocktäschel et al. (2016) employed word-by-word attention to obtain a sentence pair encoding from fine-grained reasoning via soft alignment of words and phrases in the premise and hypothesis, which achieved very promising results on the SNLI data set. Wang and Jiang (2017) proposed match LSTM for natural language inference that tries to match the current word in the hypothesis with an attention-weighted representation of the premise calculated by word-by-word attention. But these methods only consider word granularity information. Lu et al. (2020a) proposed a hierarchical encoding model (HEM) for sentence representation, further enhancing sentence interaction through a hierarchical matching mechanism. Yu R. et al. (2021) found that the available neural networks were usually limited to 1D sequential models, which hampered the performance to be improved further. Therefore, a novel neural architecture was proposed for sentence pair modeling, which utilizes 1D sentences to construct multidimensional feature maps similar to images containing multiple color channels. However, retrieval models are usually used in task-based dialogue generation and make use of only domain-specific data sets for training. In these situations, the generalization ability of the aforementioned models is poor, and they cannot respond to common input questions.

Based on the previous discussion, this study proposes a multi-granularity matching model based on Siamese networks (MGMSN). This method not only uses the deep learning method of character granularity and word granularity extraction to improve the accuracy of similarity calculation but also adds shallow semantic matching to increase the generalization of the model so that the model can still respond well to statements outside the corpus.

The rest of this article is arranged as follows. Some related work is introduced in Section 2. The architecture of the proposed MGMSN is detailed in Section 3. The experiment results of seven algorithms on the Chinese semantic similarity data set LCQMC by Liu et al. (2018) are compared in Section 4. In this section, we also detail the ablation experiments to show the effectiveness of each part of the model. Finally, we summarize this study in Section 5.

## 2 Related Work

In this section, we briefly introduce some related theories and concepts. Specifically, bidirectional LSTM (BiLSTM) will be used to extract the character granularity and word granularity features. Siamese networks will be the core components of the proposed model.

### 2.1 BiLSTM

The most important part of the text analysis process is the analysis of sentence sequences. Recurrent neural networks (RNNs) have a wide range of applications in solving sequence information problems, and their network structure is significantly different from traditional neural networks (Yu et al. (2020); Wang et al. (2022)). There will be a long-term dependency problem in the RNN learning process. This is because the connection relationship between the inputs and outputs is not ignored, resulting in forgetting the previous text information, which will cause the gradient disappearance or gradient explosion phenomenon.

**FIGURE 1:**
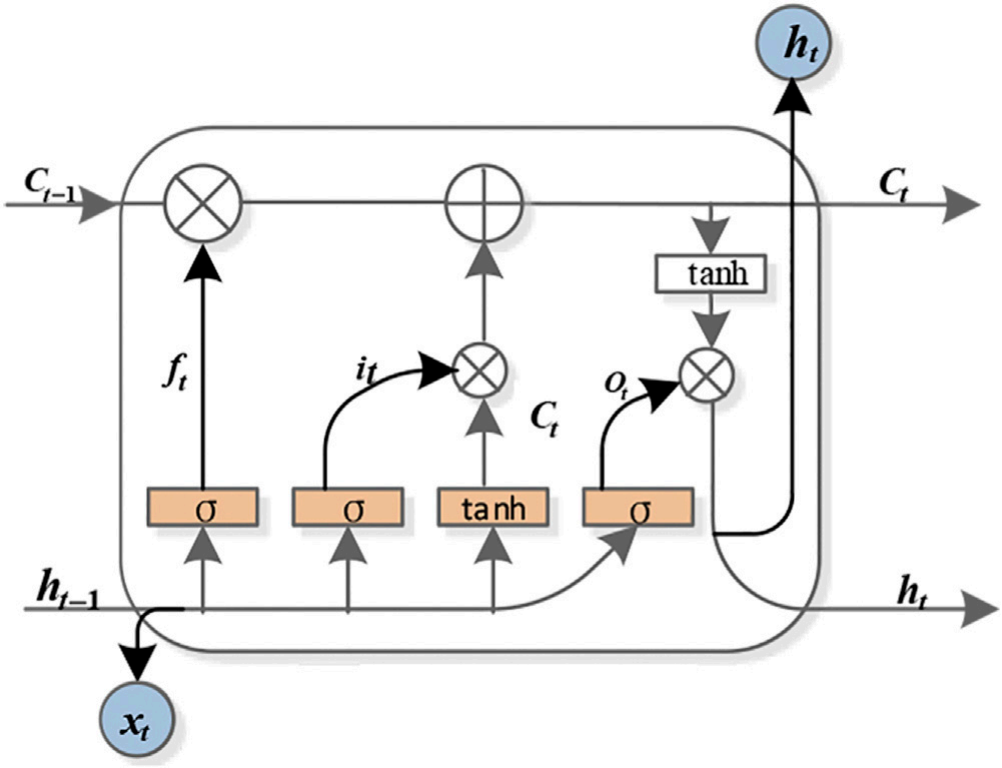
LSTM cell.

The long short-term memory network (LSTM) can solve this problem. It provides a gate mechanism to manage information to limit the amount of information and uses memory cells to store long-term historical information. Adding gates is actually a multilevel feature selection method (Na et al. (2021)). The LSTM model mainly includes input gates *i*_*t*_, forgetting gates *f*_*t*_, output gates *O*_*t*_ and memory units *C*_*t*_. The specific structure is shown in Figure 1.

In the first, LSTM must pass the forgetting gate to decide which information in the previous cell unit needs to be forgotten. It is completed by the sigmoid function, which calculates a number from 0 to 1 by receiving the weighted sum of the output at the previous time (time *t* − 1) and the input at this time (time *t*), where 0 means completely discarded and 1 means all retention. Its calculation is shown in Eq. 1:

$$f_{t}=\sigma \left(w_{f}\cdot \left[h_{t-1},x_{t}\right]+b_{f}\right).$$ (1)

After inputting the information required by the door control unit, we get

$$i_{t}=\sigma \left(w_{i}\cdot \left[h_{t-1},x_{t}\right]+b_{i}\right).$$ (2)

$$C_{t}=f_{t}\times C_{t-1}+i_{t}\times \tanh\left(w_{f}\cdot \left[h_{t-1},x_{t}\right]+b_{0}\right).$$ (3)

The information controlled by the output gate is used for the task output at this moment, and its calculation process is given as follows:

$$O_{t}=\sigma \left(w_{o}\cdot \left[h_{t-1},x_{t}\right]+b_{o}\right),$$ (4)

$$h_{t}=O_{t}\cdot \tanh\left(C_{t}\right).$$ (5)

Among them, *w*_*i*_, *w*_*f*_, and *w*_*o*_ are the weight matrices of the input gate, forgetting gate, and output gate, respectively; *b*_*i*_, *b*_*f*_, and *b*_*o*_ are the bias matrices of the input gate, forgetting gate, and output gate, respectively; *σ* is the sigmoid activation function; *h*_*t*−1_ and *h*_*t*_ represent the state of the previous hidden layer and the current hidden layer, respectively; and *x*_*t*_ represents the input of the current cell.

However, LSTM still has defects. It cannot effectively use the information after the word and cannot effectively capture weaker semantic information but can only use the information before the current word. In fact, the word semantics is related not only to the previous information but also to the information behind the word. Therefore, the text sequence is reversely integrated into the model, so that the model becomes a bidirectional long short-term memory network (BiLSTM) structure model composed of forward and reverse. The BiLSTM network takes the word vector as the model input and obtains the hidden layer state vector through the forward and backward units of the hidden layer, respectively. Considering $\vec{H}=\left(h_{1},h_{2}\ldots .h_{t}\right)$ and $\overset{⃖}{H}=\left(h_{1},h_{2}\ldots .h_{t}\right)$ as the forward and backward outputs of the hidden layer, the output of the BiLSTM hidden layer is obtained as follows:

$$H=\left(\vec{H},\overset{⃖}{H}\right).$$ (6)

### 2.2 Siamese Networks

**FIGURE 2:**
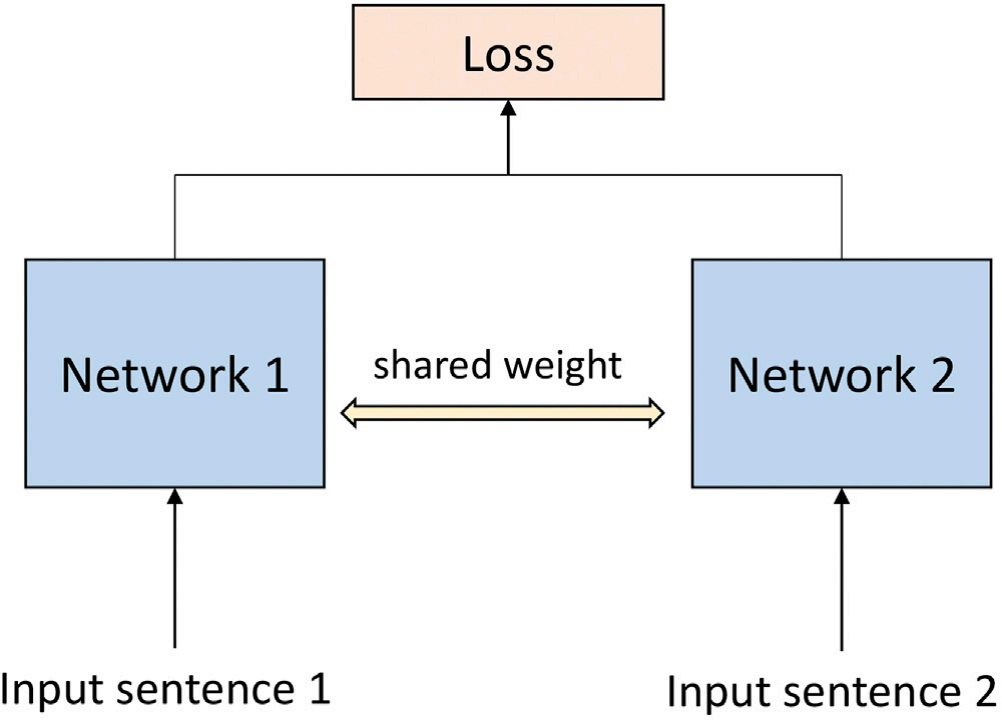
Siamese network frame diagram.

A Siamese network (Bromley et al. (1993)) is an architecture for non-linear metric learning with similarity information. It naturally learns representations that embed the invariance and selectivity desired by the explicit information about similarity between pairs of objects. In contrast, an auto-encoder (Wang et al. (2016)) learns invariance through added noise and dimensionality reduction in the bottleneck layer and selectivity through the condition that the input should be reproduced by the decoding part of the network. A Siamese network learns an invariant and selective representation directly through the use of similarity and dissimilarity information. In natural language processing, Siamese networks are usually used to calculate the semantic similarity between sentences (Kenter et al. (2016); Mueller and Thyagarajan (2016); Neculoiu et al. (2016)). The structure of the Siamese network is shown in Figure 2.

Generally, to calculate semantic similarity, sentences will be reformed as sentence pairs and then input into a Siamese network (Fan et al. (2020)).

## 3 Proposed MGMSN Model

In this section, we will introduce the proposed MGMSN model in detail. It includes two basic blocks, a deep semantic matching block and a shallow semantic matching block.

### 3.1 Deep Semantic Matching Block

For convenience, in this study, the problem of calculating the input problem and the context in the retrieval model is described as the problem of calculating the similarity between the input problem *x*_1_ and each context in the corpus *x*_2_. First, we use the tool of Jieba (Wieting et al. (2016)) for word segmentation and character segmentation. The character segmentation is to segment the sentence according to the single character. For sentences *x*_1_ and *x*_2_ that need to be calculated for similarity, after word segmentation and character segmentation, two representations of word sequence and character sequence can be obtained, respectively, which are recorded as word sequence 1, character sequence 1, word sequence 2, and character sequence 2. After finishing segmentation, the word sequence and character sequence are converted into a single vector representation through the embedding layer, and finally, the embedding matrix of the sentence is formed. The embedding layer maps each word into a vector by loading the weight of the pretrained Word2vec model.

**FIGURE 3:**
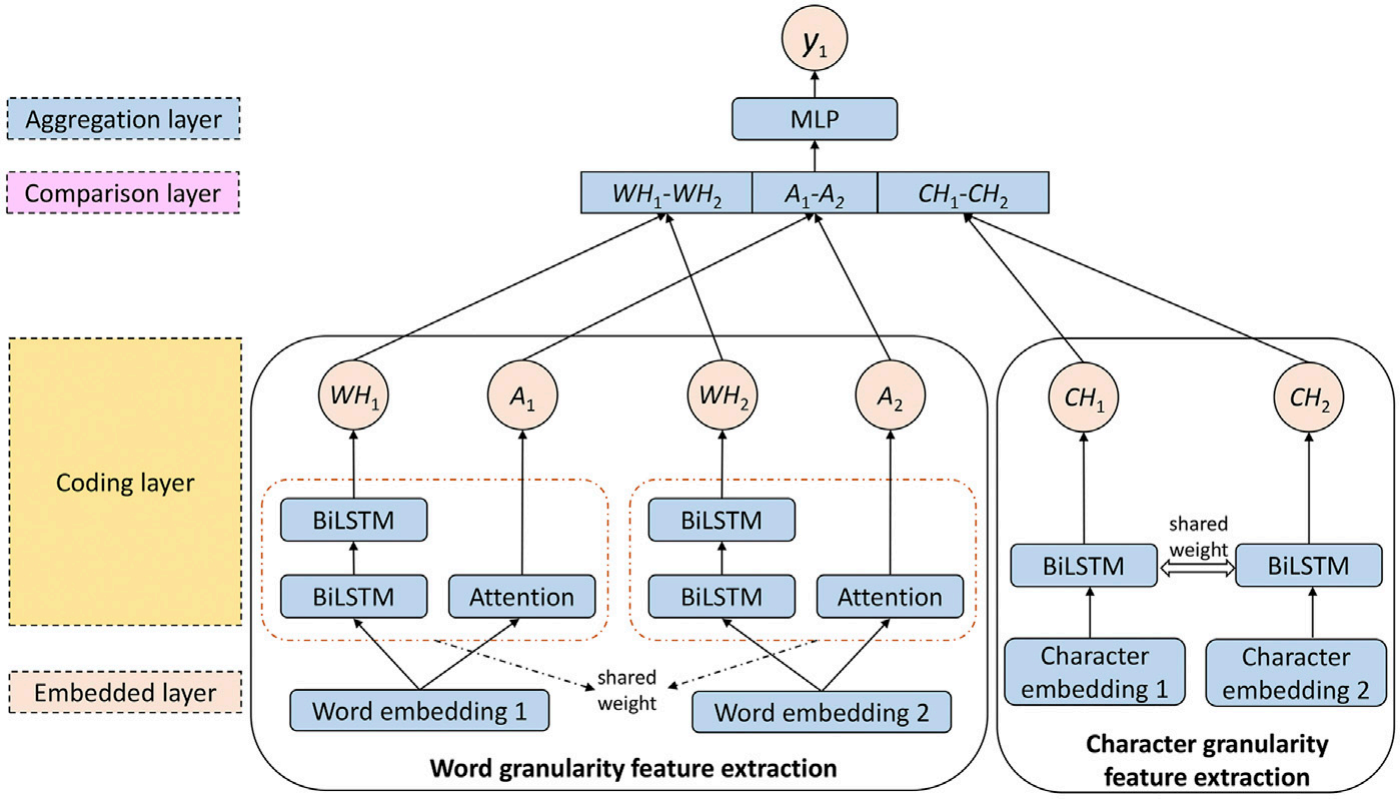
Structure diagram of the deep semantic matching algorithm.

The network structure of the deep semantic matching algorithm is mainly divided into four layers, including the embedding layer, coding layer, comparison layer, and aggregation layer. Figure 3 shows the structure of the deep semantic matching algorithm designed in this study. The deep semantic matching algorithm is mainly divided into two parts: word granularity feature extraction and character granularity feature extraction.

#### 3.1.1 Word Granularity Feature Extraction

In our deep semantic matching block, Siamese networks are used to learn the similarity between input sentences. To learn more, the BiLSTM and attention mechanisms are used to analyze and extract the semantics of sentences, and the results are combined to obtain the deep semantic matching feature of word granularity.

After tokenization, the input statements *x*_1_ and *x*_2_ are converted into word sequences *Q* and *P*, respectively. The embedding matrix of a word sequence is further obtained by the embedding layer, which is denoted as *Q* ∈ **R** ^*d*×*q*^ and *P* ∈ **R** ^*d*×*q*^, where *d* is the dimension of the word vector, and each column of *Q* and *P* represents the word vector of a word. After obtaining the embedding matrix of the input statement, the encoding layer is responsible for further feature extraction of the embedded matrix to obtain the implicit semantics of the sentence. The coding layer is the core part of the deep semantic matching algorithm. To obtain more information, in the calculation of word granularity, we use two feature extractors, BiLSTM and Attention.

In standard NLP tasks, such as text matching and named entity recognition, BiLSTM is much better than standard LSTM. The number of layers of LSTM will greatly affect the training efficiency of the model. If the LSTM has more than 3 layers, it will be difficult to train. Therefore, our proposed model adopts two-layer BiLSTM. After the embedding layer, the embedded matrix of the input statement is input into the BiLSTM for calculation, and the hidden state of the last moment of the BiLSTM is output *h*_*bi*−*lstm*_. The word embedding matrix of the input sentence *x*_1_, *x*_2_ is changed as the semantic vectors $v_{\text{word }-lstm}^{q}$ and $v_{\text{word }-lstm}^{p}$ through the BiLSTM, corresponding to *WH*_1_ and *WH*_2_ in Figure 3.

Deep neural networks can learn more information through training, but because of the disappearance of gradients, we cannot deepen the neural network infinitely. Therefore, in this study, the neural network is constructed horizontally. In addition to the feature extraction by BiLSTM, the attention layer is added to further learn the semantic information of two input statements embedded in matrices *Q* and *P*. There are many ways to realize attention mechanisms. Here, the self-attention mechanism is used for feature extraction. Its essence is to align the text to obtain more information in a targeted manner. By learning a set of weight parameters *W* ∈ *R* ^*d*^, the words embedded in the input statement is aligned to obtain the attention weight vectors $A_{Q}=\left[a_{1}^{q},a_{2}^{q},\ldots ,a_{n}^{q}\right]$ and $A_{P}=\left[a_{1}^{p},a_{2}^{p},\ldots ,a_{m}^{p}\right]$ , corresponding to *A*_1_ and *A*_2_ in Figure 3, where *n* represents the length of input statement *x*_1_, *m* represents the length of input statement *x*_2_, and the formulas of attention weight matrix are as follows,

$$A_{Q}=\mathrm{softmax}\left(\tanh\left(QW\right)\right),$$ (7)

$$A_{Q}=\mathrm{softmax}\left(\tanh\left(PW\right)\right).$$ (8)

The vectors in the embedded matrix are further weighted and summed to obtain attention semantic vectors $v_{\text{attention }}^{q}=QA_{q}$ and $v_{\text{attention }}^{p}=PA_{p}$ .

#### 3.1.2 Character Granularity Feature Extraction

At present, there is still an important problem in text matching tasks, which is the problem of out of vocabulary (OOV). There are only 2,500 Chinese characters in common use in daily life, but the number of words made up of these characters is huge. With current technology, we cannot get a word vector representation of every word. For the trained model, if the input new sentence contains untrained words, the model cannot obtain the word vector representation of untrained words, which will affect the performance of the whole model. Because of the limitation of the size of the corpus, OOV words are very easy to appear in the calculation of word granularity, and only using a model based on word granularity will reduce the discrimination ability. Therefore, in addition to calculating the similarity of the word granularity, this study further expands the granularity to the character level to obtain more text features and improve the flexibility of the model.

By adding a character granularity Siamese network, the characteristics of the text sequence can be analyzed and captured at a more fine-grained level, which further solves the problem of OOV. However, more detailed segmentation of text sequences will greatly increase the complexity of the model when capturing features. To reduce the complexity of the model and avoid the overfitting problem caused by too many parameters, only single-layer BiLSTM is used in the character granularity Siamese network. In the encoding layer, the weights in the Siamese network of word granularity and character granularity are shared.

After the tokenization, a character embedding layer is used to obtain the character embedding matrix of the input statement, and the semantic vectors $v_{char-lstm}^{q}$ and $v_{char-lstm}^{p}$ of each statement are obtained in the character granularity through the BiLSTM, corresponding to *CH*_1_ and *CH*_2_ in Figure 3.

Through coding layer calculation, three kinds of semantic vectors are obtained for each input sentence, namely, the BiLSTM semantic vector on word granularity, the attention semantic vector on word granularity, and the BiLSTM semantic vector on character granularity. These semantic vectors are further passed into the comparison layer, and each feature is combined according to the comparison function to depict the spatial difference of the input sentence. Through the element subtraction of three kinds of semantic vectors of input statement, the difference information between semantics can be obtained. The formula of the output vector *c* obtained by comparing functions is given as follows:

$$c=\left[v_{\text{word}-\text{lstm }}^{q}-v_{\text{word}-\text{lstm }}^{p};A^{q}-A^{p};v_{\text{char }-\text{lstm }}^{q}-v_{\text{char }-1\text{ stm }}^{p}\right].$$ (9)

Finally, the result of the feature comparison is passed to the aggregation layer, which is composed of a multilayer perceptron (MLP). The final output of the deep neural network is computed as follows:

$$y_{1}=\sigma \left(Wc+b\right).$$ (10)

### 3.2 Shallow Semantic Matching Algorithms

The retrieval model is more suitable for task-based conversation systems, so the collected corpus is often targeted at a specific field. Through these corpora, the neural network model is trained to learn the semantic information of sentences in the corpus and judge the similarity between sentences. Due to the limitations of the corpus domain, the similarity of sentences in a specific domain can be calculated well, but for sentences outside the corpus, it is often difficult to calculate the similarity of sentences because the neural network lacks sufficient training information and generalization ability. Following Google’s Word2vec, many companies have trained word vectors on large corpora and have opened up word vector weights. After the sentence is pretrained by Word2vec to get the word vector, the word vector of all the words in the sentence is embedded as the sentence vector, and the similarity of the input sentences is obtained by directly calculating the similarity of the two input sentence vectors. In the process of word vector training with good results, much semantic information is learned. Due to the lack of special domain knowledge and limited generalization ability, this method is even more effective in text similarity tasks than the LSTM model. By calculating the shallow semantic matching of sentences, for sentences outside the domain, the model can give a reasonable response through multilevel analysis.

The shallow semantic matching algorithm also uses the embedding layer to obtain the embedding matrices *Q* and *P* of the input statement. Although there are many ways to convert the word embedding matrix into a word vector, after comprehensive consideration, we choose word vector summation and averaging. Moreover, these two methods do not need additional parameter training and can directly obtain the embedded representation of the sentence. They can be formulated as follows:

$$v_{mean}=\frac{1}{n}\overset{n}{\underset{j}{\sum }}W_{w}^{x_{i}},$$ (11)

$$v_{sum}=\overset{n}{\underset{i}{\sum }}W_{w}^{x_{i}}.$$ (12)

**FIGURE 4:**
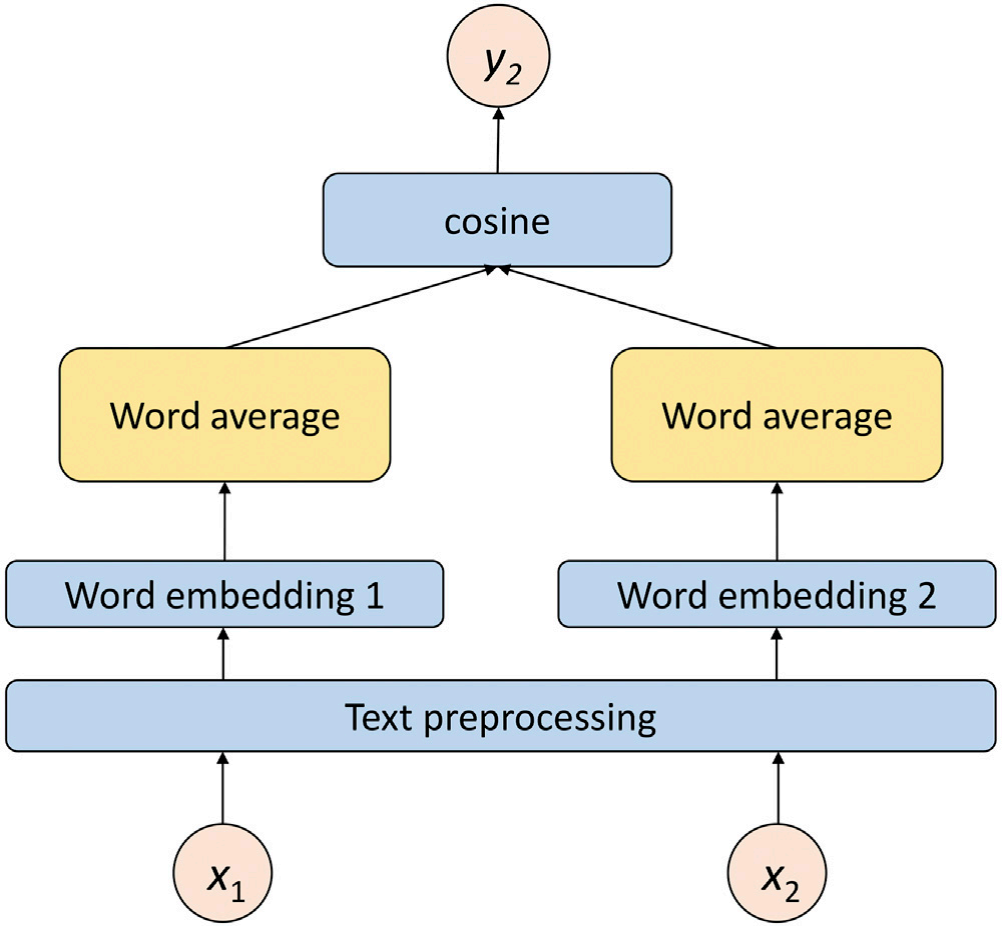
Structure diagram of shallow semantic matching algorithm.

There is no significant difference in the performance of sentence representation using word vector summation or averaging, so this study uses the word vector averaging method for semantic representation. First, the embedded matrix is averaged by word, the cosine similarity of the two average word vectors is calculated, and the similarity is taken as the output of the shallow semantic matching module. The schematic diagram of the shallow semantic matching module is given in Figure 4.

After obtaining the average word representations of sentences $v_{\text{mean }}^{q}$ and $v_{\text{mean }}^{p}$ respectively, the shallow semantic similarity *y*_2_ is obtained by calculating the cosine similarity of the two statements as follows:

$$y_{2}=\frac{v_{\text{mean }}^{q}\cdot v_{\mathrm{mean}}^{p}}{\left\|v_{\text{mecn }}^{q}\right\|\times \left\|v_{\text{mean }}^{p}\right\|}.$$ (13)

The shallow semantic matching algorithm itself has no training parameters, but the word embedding weight of the previous embedding layer is a trainable parameter. The purpose of this setting is to allow the shallow semantic matching module to “update” the training of the embedding layer.

### 3.3 Framework of MGMSN Model

The input of MGMSN is a sentence pair *X*=(*x*_1_, *x*_2_), and its output *y* is the similarity score of the sentences *x*_1_ and *x*_2_. After obtaining the deep semantic similarity *y*_1_ and shallow semantic similarity *y*_2_ of the two sentences, respectively, the final output neuron is achieved by combining these two different levels of similarity.

$$y=\sigma \left(w_{1}y_{1}+w_{2}y_{2}+b\right).$$ (14)

Here, *w*_1_, *w*_2_, and *b* are the weight parameters of the neural network. The training goal is to minimize the cross-entropy between the predicted value and the real value, which is given by

$$\text{ loss }=-\overset{N}{\underset{i=1}{\sum }}\left(y_{i}\log p_{i}+\left(1-y_{i}\right)\log\left(1-p_{i}\right)\right),$$ (15)

where *y*_*i*_ is the real value and *p*_*i*_ is the predicted value.

**FIGURE 5:**
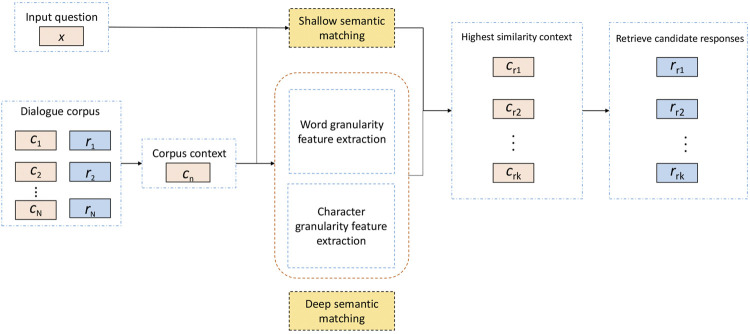
Structure of MGMSN.

Through the processing of the above two parts, we get the similarity of the two input sentences. When applied to the conversation model, it uses the text matching model to calculate the matching degree between the input question and each context in the corpus. The higher the matching degree between the input question and the context is, the more appropriate the response corresponding to the context will be. This method increases the accuracy of text similarity calculations to a certain extent through multilevel and multi-granularity calculations. Combining Figure 3 and Figure 4, the structure diagram of MGMSN is shown in Figure 5.

## 4 Experiment Results and Analysis

To evaluate the effectiveness of the proposed model, we compare MGMSN with six current mainstream text matching algorithms, they are Deep Structured Semantic Model (DSSM) (Huang et al. (2013)), Siamese LSTM network (Siamese LSTM) (Neculoiu et al. (2016)), Attention-Based Convolutional Neural Network for Modeling Sentence Pairs (ABCNN) (Yin et al. (2016)), Enhance Sequential Inference Model (ESIM) (Greff et al. (2016)), Deep Interactive Text Matching (DITM) model (Yu C. et al. (2021)), and Frame-based Multi-level Semantics Representation (FMSR) model (Guo et al. (2021)).

### 4.1 Data Description

In this study, we use the open Chinese semantic similarity data set LCQMC (Liu et al. (2018)) for training. The LCQMC is a semantic matching data set published by the Harbin Institute of Technology in COLING 2018. The task is to judge whether the semantics of two questions are similar. The LCQMC data set contains 260,086 annotated data points. By dividing different fields and extracting the most relevant question set from Baidu QA, the LCQMC data set is manually filtered and annotated after preliminary screening. In the LCQMC corpus, the maximum length of sentences is 131 characters, the shortest length is 2 characters, and the average length of sentences is 10 characters, which belongs to the category of short text. The data set is predivided into a training set, a validation set, and a test set, and their descriptions are shown in Table 1.

**TABLE 1:** Data set structure.

Data	Positive sample size	Negative sample size	Total
Training set	138,574	100,192	238,766
Validation set	4,402	4,400	8,802
Test set	6,250	6,250	12,500

### 4.2 Implementation Details and Parameter Settings of MGMSN

To prevent the model from overfitting, we apply techniques such as dropout (Baldi and Sadowski (2013)), early stopping (Prechelt (1998)), and batch normalization (Ioffe and Szegedy (2015)). A dropout layer is added between the LSTM recurrent layer and the deep semantic matching aggregation layer, the function of similar data enhancement is increased to a certain extent during training by dropping a certain proportion of neurons randomly, while the co-adaptation relationship between neurons is reduced to prevent overfitting. Early stopping is a common method to prevent model overfitting. The data set is divided into training sets, validation sets, and test sets, and the training process is monitored through the validation sets. In the process of model training, the validation set is used to monitor the training accuracy (it can also be the training loss), and when the specified conditions are satisfied, the training process will be terminated in advance. In this study, the tolerance set in the experiment is 2, that is, if the accuracy of the two training process validation sets is not improved, the training process will be terminated in advance.

During the training of the neural network, as the parameters of the previous layers change, the input data distribution of the current layer will also change accordingly. Therefore, the current layer needs to be continuously updated to adapt to the new data distribution (Tang et al. (2018)). Batch normalization normalizes the input of each layer of the network to make the output obey the normal distribution with a mean value of 0 and a variance of 1, to avoid the problem of variable distribution deviation. The batch normalization layer is applied between multilayer perceptrons. The NAdam method (Kingma and Ba (2015)) is selected as the model optimization strategy. Table 2 shows the parameter settings of the model in the experimental environment. All word vectors are pretrained word vectors and updated during the training process.

**TABLE 2:** Model parameter setting.

Parameter	Value
Word vector dimension	300
Perceptron dimension	128
LSTM hidden state dimension	128
LSTM Circulation layer Dropout	0.5
Dropout	0.5
Maximum length of word input	20
Maximum length of character input	40
Word Siamese LSTM hidden state dimension	32
Maximum number of iterations	15

### 4.3 Comparison Results of Sentence Matching Algorithms

The proposed MGMSN model will be evaluated in calculating the accuracy between sentences with the other six sentence matching models. Table 3 shows the results of these models.

**TABLE 3:** Comparison of matching accuracy of different models.

Model	Embedded type	Training set	Validation set	Test set
DSSM	Char	0.66	0.59	0.63
Siamese LSTM	Word	0.91	0.78	0.77
ABCNN	Char	0.90	0.76	0.79
ESIM	Word	0.77	0.67	0.72
DITM	Word	0.81	0.74	0.77
FMSR	Word	0.92	0.83	0.79
MGMSN	Word/Char	0.94	0.84	0.85

In the table, “Char” means embedding at character granularity, and “Word” means embedding at word granularity. It can be seen that although DSSM uses a fine-grained character-based embedding method, the comprehensive matching effect is poor because it only uses the fully connected layer for feature extraction. In the training set, the ESIM model only has an accuracy of 0.77 on the training set, which shows that the ESIM model does not fit the data set well, and the role of text interactive reasoning information in text matching does not have an obvious effect. The under-fitting state of the ESIM model on the training set causes the model to perform worse than the Siamese LSTM model on the validation set and test set. In contrast, although the Siamese LSTM model and the ABCNN model can achieve an effect of more than 0.9 in the training set, their effect in the validation set and test set is greatly reduced. Since the DITM model is able to perform multiple iterations of the interaction process, it can obtain deep interaction information and extract the relationship between text pairs through multi-view pooling. Therefore, the model can achieve 81% accuracy on the training set, which is about 4% higher than the ESIM model. However, the Siamese network has irreplaceable advantages, and the accuracy of the Siamese LSTM model at the word level is about 10% higher than that of the DITM model in the training set. The FMSR model exploits frame knowledge to explicitly extract multilevel semantic information in sentences for text matching tasks. The accuracy of the FMSR model is higher than the aforementioned models in the training set, validation set, and test set. However, the model still has shortcomings, the training set accuracy is about 0.02 lower than the MGMSN model, the validation set is about 0.01 lower, and the test set is about 0.06 lower.

### 4.4 Ablation Experiments

In this subsection, we conduct a set of ablation experiments on the model to prove the effectiveness of each component of the MGMSN model. Specifically, we sequentially remove the character granularity Siamese network in the component, attention feature extraction, and shallow semantic matching block. The changes in matching accuracy of the ablation experiments are given in Table 4.

**TABLE 4:** Results of ablation experiment.

	Training set	Validation set	Test set
Character granularity Siamese network removed	−0.2%	−0.5%	−0.9%
Attention feature extraction removed	−0.1%	−1.5%	±0.5%
Shallow semantic matching block removed	+0.5%	+0.6%	−1.6%

It can be found that the shallow semantic matching block has the greatest impact on the accuracy of the final test set. After deleting it, the performance of the entire model in the test set dropped by 1.6%. At the same time, the accuracy of the training set and the validation set is improved, which means that the data distribution of the training set and the validation set is relatively consistent. However, the accuracy of the test set dropped sharply, which indicates that the data distribution of the test set and the training set may be inconsistent, and the model could not be generalized well to the test set. This further shows that the shallow semantic matching block improves the generalization ability of the model to a certain extent.

After removing the character granularity Siamese network, it can be found that the accuracy of the MGMSN model decreases to varying degrees on the training set, validation set, and test set. In the test set, the performance of the model decreases by 0.9%, which shows that the character granularity Siamese network can not only solve the problem of OOV but also extract different granularities of text semantic information to a certain extent and improve the accuracy of model matching.

When the attention feature extraction part is removed, the training process has little effect on the model, and the training set fitting accuracy only decreases by 0.1%, which indicates that the Siamese network structure is suitable for text semantic matching tasks. However, the performance of the validation set is reduced by 1.5%, which shows that the model has a certain degree of overfitting. In the final test set, we find that the performance of the test set is very unstable, and the performance influence fluctuates within the range of (−0.5% to 0.5%), which also means that the addition of attention to extracting features improves the robustness of the model.

Due to the real-time requirements of the conversation system, we tested the prediction time of the MGMSN model under the configuration of the CPU model i5-8250U and memory of 16G. The experiment shows that the real-time prediction time of the MGMSN model is 2 ms, which can meet the demand of millisecond-level response. It can be seen that even in the CPU environment, the MGMSN model proposed in this article can fully meet the needs of real-time conversation.

## 5 Conclusion and Future Work

Different from other models that only use deep neural network to improve sentence matching similarity, in order to further improve the accuracy and generalization ability of the model, we not only optimize the deep neural network but also combine it with the traditional matching model. In this way, the model responds well to user input questions whether or not they are in the trained corpus.

The multi-granularity matching model based on the Siamese network proposed in this article improves the scalability, robustness, and accuracy ability of the model by combining deep semantic matching and shallow semantic matching algorithms and using attention and BiLSTM to extract features in parallel to obtain matching information from different views. To solve the problem of OOV, the character granularity Siamese structure is further added to the deep semantic matching to enrich the network structure and obtain fine-grained matching features. The ablation experiments show that the character granularity Siamese network, attention feature extraction, and shallow semantic matching algorithms all contribute to the MGMSN model. Experiments show that the accuracy of the MGMSN model proposed in this article is higher than that of the other six current mainstream text matching algorithms.

Although the multi-granularity compound conversation model based on the Siamese network proposed in this article has excellent performance, there is still room for further improvement in terms of practical problems.

1) Most of the conversations are more than single round. How to analyze and respond to multi-round conversation is very important. The model proposed in this article is mainly for a single-round conversation. For multi-round conversation, it cannot perform multi-sentence contextual analysis and maintain the consistency of responses. Therefore, in future work, a hierarchical structure can be added to capture the semantics of sentences and the semantics of multiple rounds of context at the same time, to improve the model’s response accuracy and topic consistency.
2) It is very natural for humans to express emotional language. But how the conversation system can capture emotions and express them in real time is still a challenge. Therefore, in future work, we can consider the emotional analysis of the input sentences and use the emotional dictionary to generate the emotional response so that the response sentence is more like a real person’s response and ensure the continuity of the conversation.

## Data Availability

The original contributions presented in the study are included in the article/Supplementary Material; further inquiries can be directed to the corresponding author.

## References

[B1] Baldi P. Sadowski P. J. (2013). Understanding Dropout. Adv. Neural Inf. Process. Syst. 26, 2814–2822.

[B2] Bowman S. R. Angeli G. Potts C. Manning C. D. (2015). A Large Annotated Corpus for Learning Natural Language Inference. arXiv preprint arXiv:1508.05326. 10.18653/v1/d15-1075

[B3] Bromley J. Bentz J. W. Bottou L. Guyon I. LeCun Y. Moore C. (1993). Signature Verification Using a “Siamese” Time Delay Neural Network. Int. J. Patt. Recogn. Artif. Intell. 07, 669–688. 10.1142/s0218001493000339

[B4] Colby K. M. (1981). Modeling a Paranoid Mind. Behav. Brain Sci. 4, 515–534. 10.1017/s0140525x00000030

[B5] Fan Z. Song X. Chen Q. Jiang R. Shibasaki R. Tsubouchi K. (2020). Trajectory Fingerprint: One-Shot Human Trajectory Identification Using Siamese Network. CCF Trans. Pervasive Comp. Interact. 2, 113–125. 10.1007/s42486-020-00034-2

[B6] Greff K. Srivastava R. K. Koutník J. Steunebrink B. R. Schmidhuber J. (2016). Lstm: A Search Space Odyssey. IEEE Trans. Neural Netw. Learn. Syst. 28, 2222–2232. 10.1109/TNNLS.2016.2582924 27411231

[B7] Guo S. Guan Y. Li R. Li X. Tan H. (2021). Frame-based Multi-Level Semantics Representation for Text Matching. Knowledge-Based Syst. 232, 107454. 10.1016/j.knosys.2021.107454

[B8] Huang P.-S. He X. Gao J. Deng L. Acero A. Heck L. (2013). “Learning Deep Structured Semantic Models for Web Search Using Clickthrough Data,” in Proceedings of the 22nd ACM international conference on Information & Knowledge Management, 2333–2338. 10.1145/2505515.2505665

[B9] Ioffe S. Szegedy C. (2015). “Batch Normalization: Accelerating Deep Network Training by Reducing Internal Covariate Shift,” in International conference on machine learning (PMLR), 448–456.

[B10] Kenter T. Borisov A. de Rijke M. (2016). Siamese CBOW: Optimizing Word Embeddings for Sentence Representations. Stroudsburg, Pennsylvania, USA: The Association for Computer Linguistics.

[B11] Kingma D. P. Ba J. (2015). “Adam: A Method for Stochastic Optimization,” in 3rd International Conference on Learning Representations, ICLR 2015, San Diego, CA, USA, May 7-9, 2015, Conference Track Proceedings. Editors Bengio Y. LeCun Y..

[B12] Liu X. Chen Q. Deng C. Zeng H. Chen J. Li D. (2018). “Lcqmc: A Large-Scale Chinese Question Matching Corpus,” in Proceedings of the 27th International Conference on Computational Linguistics, 1952–1962.

[B13] Lu W. Yu R. Wang S. Wang C. Jian P. Huang H. (2021). Sentence Semantic Matching Based on 3d Cnn for Human-Robot Language Interaction. ACM Trans. Internet Technol. 21, 1–24. 10.1145/3450520

[B14] Lu W. Zhang X. Lu H. Li F. (2020a). Deep Hierarchical Encoding Model for Sentence Semantic Matching. J. Vis. Commun. Image Representation 71, 102794. 10.1016/j.jvcir.2020.102794

[B15] Lu W. Zhang Y. Wang S. Huang H. Liu Q. Luo S. (2020b). Concept Representation by Learning Explicit and Implicit Concept Couplings. IEEE Intell. Syst. 36, 6–15.

[B16] Ma T. Wang H. Zhang L. Tian Y. Al-Nabhan N. (2021a). Graph Classification Based on Structural Features of Significant Nodes and Spatial Convolutional Neural Networks. Neurocomputing 423, 639–650. 10.1016/j.neucom.2020.10.060

[B17] Ma T. Zhou H. Tian Y. Al-Nabhan N. (2021b). A Novel Rumor Detection Algorithm Based on Entity Recognition, Sentence Reconfiguration, and Ordinary Differential Equation Network. Neurocomputing 447, 224–234. 10.1016/j.neucom.2021.03.055

[B18] Mueller J. Thyagarajan A. (2016). Siamese Recurrent Architectures for Learning Sentence Similarity. Palo Alto, California, U.S.: AAAI Press, 2786–2792.

[B19] Na H. Shin Y. Lee D. Lee J. (2021). Lstm-based Throughput Prediction for Lte Networks. ICT Express. 10.1016/j.icte.2021.12.001

[B20] Neculoiu P. Versteegh M. Rotaru M. (2016). “Learning Text Similarity with Siamese Recurrent Networks,” in Proceedings of the 1st Workshop on Representation Learning for NLP, 148–157. 10.18653/v1/w16-1617

[B21] Prechelt L. (1998). Automatic Early Stopping Using Cross Validation: Quantifying the Criteria. Neural Networks 11, 761–767. 10.1016/s0893-6080(98)00010-0 12662814

[B22] Rocktäschel T. Grefenstette E. Hermann K. M. Kociský T. Blunsom P. (2016). Reasoning about Entailment with Neural Attention. arXiv:1509.06664v4.

[B23] Shang L. Lu Z. Li H. (2015). Neural Responding Machine for Short-Text Conversation. Stroudsburg, Pennsylvania, USA: The Association for Computer Linguistics, 1577–1586.

[B24] Shen D. Wang G. Wang W. Min M. R. Su Q. Zhang Y. (2018). Baseline Needs More Love: On Simple Word-Embedding-Based Models and Associated Pooling Mechanisms. arXiv preprint arXiv:1805.09843. 10.18653/v1/p18-1041

[B25] Tang S. Shen C. Wang D. Li S. Huang W. Zhu Z. (2018). Adaptive Deep Feature Learning Network with Nesterov Momentum and its Application to Rotating Machinery Fault Diagnosis. Neurocomputing 305, 1–14. 10.1016/j.neucom.2018.04.048

[B26] Wang J. Li X. Li J. Sun Q. Wang H. (2022). Ngcu: A New Rnn Model for Time-Series Data Prediction. Big Data Res. 27, 100296. 10.1016/j.bdr.2021.100296

[B27] Wang S. Jiang J. (2017). “A Compare-Aggregate Model for Matching Text Sequences,” in 5th International Conference on Learning Representations, ICLR 2017, Toulon, France, April 24-26, 2017, Conference Track Proceedings (OpenReview.net).

[B28] Wang Y. Yao H. Zhao S. (2016). Auto-encoder Based Dimensionality Reduction. Neurocomputing 184, 232–242. 10.1016/j.neucom.2015.08.104

[B29] Weizenbaum J. (1983). ELIZA - a Computer Program for the Study of Natural Language Communication between Man and Machine. Commun. ACM 26, 23–28. 10.1145/357980.357991

[B30] Wieting J. Bansal M. Gimpel K. Livescu K. (2016). “Towards Universal Paraphrastic Sentence Embeddings,” in 4th International Conference on Learning Representations, ICLR 2016, San Juan, Puerto Rico, May 2-4, 2016, Conference Track Proceedings.

[B31] Wilensky R. (1987). “The berkeley Unix Consultant Project,” in Wissensbasierte Systeme (Berlin, Germany: Springer), 286–296. 10.1007/978-3-642-88719-2_25

[B32] Yang Y. Yih W.-t. Meek C. (2015). “Wikiqa: A challenge Dataset for Open-Domain Question Answering,” in Proceedings of the 2015 Conference on Empirical Methods in Natural Language Processing, 2013–2018. 10.18653/v1/d15-1237

[B33] Yin W. Schütze H. Xiang B. Zhou B. (2016). Abcnn: Attention-Based Convolutional Neural Network for Modeling Sentence Pairs. Tacl 4, 259–272. 10.1162/tacl_a_00097

[B34] Yu C. Xue H. Jiang Y. An L. Li G. (2021a). A Simple and Efficient Text Matching Model Based on Deep Interaction. Inf. Process. Manage. 58, 102738. 10.1016/j.ipm.2021.102738

[B35] Yu R. Lu W. Lu H. Wang S. Li F. Zhang X. (2021b). Sentence Pair Modeling Based on Semantic Feature Map for Human Interaction with Iot Devices. Int. J. Machine Learn. Cybernetics. 10.1007/s13042-021-01349-x

[B36] Yu W. Kim I. Y. Mechefske C. (2020). An Improved Similarity-Based Prognostic Algorithm for Rul Estimation Using an Rnn Autoencoder Scheme. Reliability Eng. Syst. Saf. 199, 106926. 10.1016/j.ress.2020.106926

[B37] Zhang P. Huang X. Wang Y. Jiang C. He S. Wang H. (2021). Semantic Similarity Computing Model Based on Multi Model fine-grained Nonlinear Fusion. IEEE Access 9, 8433–8443. 10.1109/ACCESS.2021.3049378

